# Glial Activation Markers in CSF and Serum From Patients With Primary Progressive Multiple Sclerosis: Potential of Serum GFAP as Disease Severity Marker?

**DOI:** 10.3389/fneur.2019.00280

**Published:** 2019-03-26

**Authors:** Ahmed Abdelhak, Tilman Hottenrott, Estrella Morenas-Rodríguez, Marc Suárez-Calvet, Uwe K. Zettl, Christian Haass, Sven G. Meuth, Sebastian Rauer, Markus Otto, Hayrettin Tumani, André Huss

**Affiliations:** ^1^Department of Neurology, University Hospital of Tuebingen, Tuebingen, Germany; ^2^Department of Neurology, University Hospital of Ulm, Ulm, Germany; ^3^Department of Neurology, University Hospital of Freiburg, Freiburg, Germany; ^4^German Center for Neurodegenerative Diseases (DZNE), Munich, Germany; ^5^BioMedical Center (BMC), Ludwig-Maximilians-Universität München, Munich, Germany; ^6^Barcelonaβeta Brain Research Center (BBRC), Pasqual Maragall Foundation, Barcelona, Spain; ^7^Neuroimmunological Section, Department of Neurology, University Hospital of Rostock, Rostock, Germany; ^8^Munich Cluster for Systems Neurology (SyNergy), Munich, Germany; ^9^Department of Neurology, University Hospital of Muenster, Münster, Germany; ^10^Specialty Hospital Dietenbronn, Schwendi, Germany

**Keywords:** SiMoA, GFAP, PPMS, glial activation, progressive multiple sclerosis, neurofilaments, CHI3L1, sTREM2

## Abstract

**Background:** In progressive multiple sclerosis (MS), glial activation is thought to be a relevant mechanism of disability progression. Therefore, *in vivo* assessment of the glial cell activity is, in the emerging treatment era of primary progressive MS (PPMS), more important than ever.

**Objectives:** To test the association of cerebrospinal fluid (CSF) and serum markers of glial activation in PPMS patients; including glial fibrillary acidic protein (GFAP), chitinase-3-like protein 1 (CHI3L1), soluble variant of triggering receptor expressed on myeloid cells 2 (sTREM2), and marker of neuroaxonal damage (Neurofilament light chain, NfL) as well as clinical severity.

**Methods:** CSF and serum samples from PPMS patients were collected in the MS-centers at Universities of Freiburg (*n* = 49), Ulm (*n* = 27), Muenster (*n* = 11), and Rostock (*n* = 6). sTREM2 and CHI3L1 levels were measured using the previously reported ELISA assays, while NfL and GFAP were measured using SIMOA assays. Clinical data included age, gender, disease duration, treatment status, and Expanded Disability Status Scale (EDSS).

**Results:** 93 CSF samples and 71 matching serum samples were analyzed. The median age of patients was 49 years and disease duration 4.5 years. GFAP_serum_ correlated with EDSS after correction for age (β = 0.3, *p* = 0.001). Furthermore, EDSS was higher in patients with a GFAP_serum_ level ≥ 151.7 pg/ml compared to patients with GFAP_serum_ below this cut-off (5.5 vs. 4.0, *p* = 0.009). Other markers did not correlate with the clinical severity. Moreover, we found a correlation between NfL_CSF_ and GFAP_CSF_, sTREM2 and CHI3L1 (ρ = 0.4 for GFAP_CSF_ and sTREM2, ρ = 0.3 for CHI3L1, *p* < 0.01 for sTREM2 and CHI3L1 and <0.001 for GFAP_CSF_). CHI3L1 did not correlate with GFAP_CSF_ but with sTREM2 (ρ = 0.4, *p* < 0.01).

**Discussion:** The correlation between the glial activation markers in CSF with the markers of neuroaxonal demise supports the notion of the glial involvement in PPMS. The positive correlation between GFAP_CSF_ with disease duration and GFAP_serum_ with the clinical severity of the disease may highlight a particular role of the astrocytes in PPMS and mark the potential of GFAP_serum_ as a disease severity marker.

## Introduction

The pathophysiology of primary progressive multiple sclerosis (PPMS) is complex and involves various mechanisms including inflammatory triggered demyelination, activation of B and T lymphocytes, mitochondrial dysfunction, and iron accumulation (1). However, the glial activation is considered to play a decisive role in the progression of neuroaxonal demise (2–4). While clinical and many radiological parameters can detect the final pathway of those different pathophysiological processes (progression of clinical disability, new MRI-lesions etc.), several other aspects such as role, extent and contribution of the various pathophysiological mechanisms remain widely unexplored. A biomarker-based approach may offer a unique window to assess such disease processes *in vivo* (5, 6). Over the last years, the level and clinical meaning of different biomarkers in CSF like glial fibrillary acidic protein (GFAP) as a marker for astrocytic activation (7–16), chitinase 3 like 1 protein (CHI3L1) (13, 14, 17–19) and soluble triggering receptor expressed on myeloid cells 2 (sTREM2) for microglial activation (20–23) and neurofilaments light chain (NfL) for neuroaxonal damage were reported. The single molecular assay (SIMOA) enables the detection of the ultra-low concentration of some of those biomarkers in serum (16, 24, 25). We previously showed that GFAP in serum correlates with the Expanded Disability Status Scale (EDSS) specifically in PPMS but not in patients with a relapsing-remitting disease course (16). Similar results were reported later from other groups (26). In this study, we aim to reproduce these findings in a large cohort of PPMS patients and to explore the clinical meaning of the other glial activation markers in PPMS.

## Methods

### Patient Selection

CSF and serum samples from patients with PPMS were collected from the University Hospitals of Freiburg, Ulm, Muenster, and Rostock. The patients were admitted or seen within the Outpatient Departments between 2010 and 2018. In all patients, the diagnosis has been revised according to the McDonald criteria from 2017 (27) after careful exclusion of relevant differential diagnoses. The lumbar puncture was performed as a part of the diagnostic workup. The clinical severity was measured by assessing the Expanded Disability Status Scale (EDSS), Multiple Sclerosis Severity Score (MSSS) as well as the Age-related Multiple Sclerosis Severity Score (ARMSS) as reported recently (28).

### CSF and Serum Sample Processing

A standardized protocol for CSF and serum collection was applied as previously recommended (29). Biosamples from patients were stored according to the predefined standard operating procedure (SOPs) at a local biobank at minus 80°C. Later they were transferred for measurement on dry ice to the biobank of the coordinating center in Ulm for further analysis. Hemolytic CSF specimens were excluded. From some patients, only CSF samples were available, with no matching serum samples.

### Assessments of the Biomarkers

GFAP and NfL in CSF and serum were measured using Simoa assays (GFAP Discovery kits and NfL Early Access assays, Quanterix Corporation). CHI3L1 was measured using the commercial ELISA-Kits (Human Chitinase 3-like 1 Quantikine ELISA Kit DC3L10, R&D Systems). sTREM2 was measured using the previously reported ELISA using the MSD Platform (21). Samples were diluted, as recommended by the manufacturer, and concentrations were calculated using the corresponding standard curve. The intra-assay coefficient of variation (CV) was assessed by measuring a QC of serum and CSF sample in 5 replicates with a CV below 10% was obtained, whereas a CV of lower than 10% had to be achieved for a valid analysis. We did not find an influence of up to 5 freeze-thaw cycles on the investigated biomarkers, except for GFAP in CSF. Here, the concentration decreased by over 50% after within 2 freeze-thaw cycles. Therefore, GFAP CSF levels between centers were compared, and exceedingly low values were excluded from the analysis.

To compare potentially pathological serum biomarker levels, we determined a cut-off in a group of 20 patients with other non-inflammatory neurological diseases we previously published (16). As the concentration of serum GFAP in a normal or healthy population is not described, we used the 90th percentile to determine a cut-off value for further analysis. This yielded a cut-off value of 151.7 pg/ml for serum GFAP. A cut-off value for serum NfL of 16 pg/ml was suggested recently (30).

### Statistical Analysis

All statistical tests were performed using SPSS® Statistics version 25 (IBM Corporation). The Shapiro-Wilk test was used to examine the distribution of the data. Mann-Whitney *U* test and Kruskal-Wallis test were used to compare medians in skewed distributed parameters. A multiple linear regression model and univariant general linear model was applied to account for a possible confounding bias caused by the strong correlation between GFAP levels and age. The Spearman's rho test was used to test for correlations. A *p*-value ≤ 0.05 was considered as statistically significant. Figures were made using GraphPad Prism 6 software (GraphPad Software Inc., La Jolla, CA, USA).

## Results

### Clinical Characteristics

A Total of 93 CSF and 71 matching serum samples were collected. The summary of the clinical characteristics is mentioned in Table 1, and the concentration of the different CSF and serum biomarkers are shown in Table 2. Fifty-five patients did not receive any disease modifying treatment at and before the time of sample collection. Thirty-eight patients were on a treatment: Mitoxantrone (*n* = 19), three monthly pulse-steroid (*n* = 11), Rituximab (*n* = 5), cyclosporine (*n* = 2), and Interferon beta 1a (*n* = 1).

**Table 1:** Clinical characteristics of the included subjects.

	**Median (25–75 percentile), *n* = 93**
Age	49 years (44–57)
Gender ♀:♂	1.1:1
Disease duration in years	4.5 (2–12)
Expanded disability status scale (EDSS) at the time of lumbar puncture (LP)	4.5 (3.5–6.5)
Multiple sclerosis severity score (MSSS)	8.1 (6.4–9.1)
Age-related multiple sclerosis severity score (ARMSS)	6.2 (4.6–7.9)

**Table 2:** Concentrations of the assessed biomarkers in CSF and serum.

	**Median (25–75 percentile)**
Cerebrospinal fluid glial fibrillary acidic protein (GFAP_CSF_) in pg/ml (*n* = 76)	7,820 (5,050–1,1165)
Serum glial fibrillary acidic protein (GFAP_serum_) in pg/ml (*n* = 71)	126.0 (104.5–174.0)
Cerebrospinal fluid neurofilaments light chain (NfL_CSF_) in pg/ml (*n* = 93)	1230.8 (840–2,125)
Serum neurofilaments light chain (NfL_serum_) in pg/ml (*n* = 71)	18.5 (12.3–25.9)
Cerebrospinal fluid chitinase 3 like 1 protein (CHI3L1) in ng/ml (*n* = 93)	210.8 (138.5–291.0)
Cerebrospinal fluid soluble triggering receptor expressed on myeloid cells 2 (sTREM2) in ng/ml (*n* = 87)	3.1 (2.3–4.4)

### Comparison Between Centers

**Figure 1:**
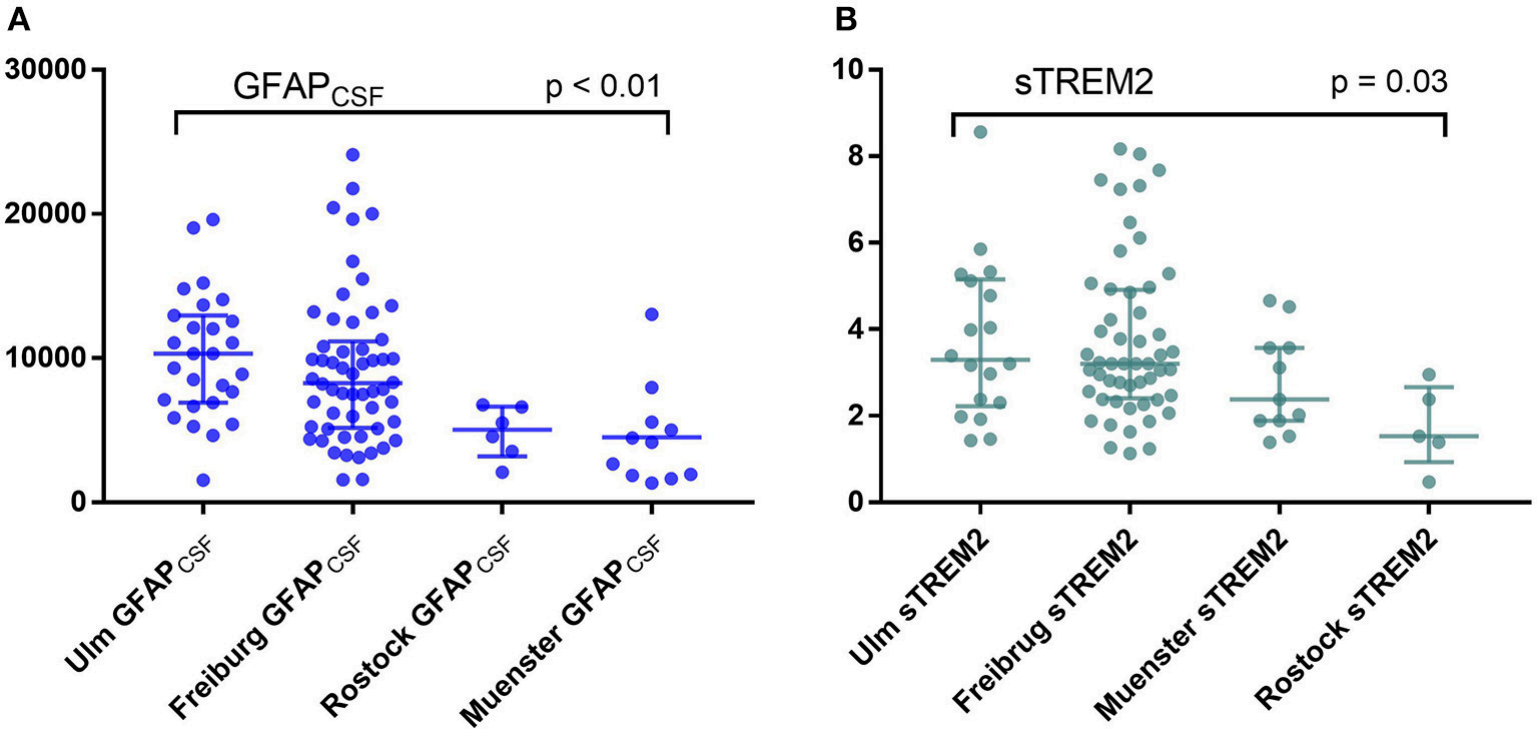
Comparison between the levels of **(A)** glial fibrillary acidic protein (GFAP) and **(B)** soluble triggering receptor expressed on myeloid cells 2 (sTREM2) in cerebrospinal fluid (CSF) between the four participating centers (Kruskal-Wallis test).

Despite having similar patients characteristics (age, gender distribution, disease duration, and disease severity as assessed by EDSS), GFAP_CSF_ levels differ between the centers; values form Muenster and Rostock were significantly lower than those from Ulm and Freiburg, whereas sTREM2 levels were lower only in the samples form Rostock compared to all other centers (Figure 1). Thus, in the following analysis, we excluded the GFAP_CSF_ from Muenster and Rostock (*n* = 17), and sTREM2 measurements form Rostock (*n* = 6). Concentrations of CHI3L1, NfL_CSF_, NfL_serum_, and GFAP_serum_ in the samples of Muenster and Rostock were included in the statistical analysis.

### Clinical Aspects

**Figure 2:**
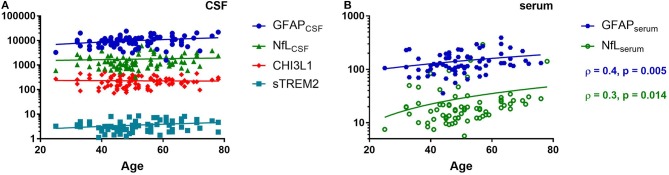
Spearman correlation of various cerebrospinal fluid (CSF) **(A)** and serum **(B)** biomarkers with the age of the patients. Glial fibrillary acidic protein (GFAP), neurofilaments light chain (NfL), chitinase 3 like 1 (CHI3L1), soluble triggering receptor expressed on myeloid cells 2 (sTREM2), ρ (Spearman rho).

None of the biomarkers correlated with the age except GFAP_serum_ and NfL_serum_ (ρ = 0.4 and 0.3, *p* = 0.005 and 0.014, respectively) (Figure 2). Gender and treatment status did not influence the levels any of the tested markers neither in CSF nor in serum (data not shown).

Of all the assessed markers (in CSF and in serum), only GFAP_CSF_ correlated with disease duration (ρ = 0.3, *p* = 0.014). None of the CSF markers correlated with disease severity.

**Figure 3:**
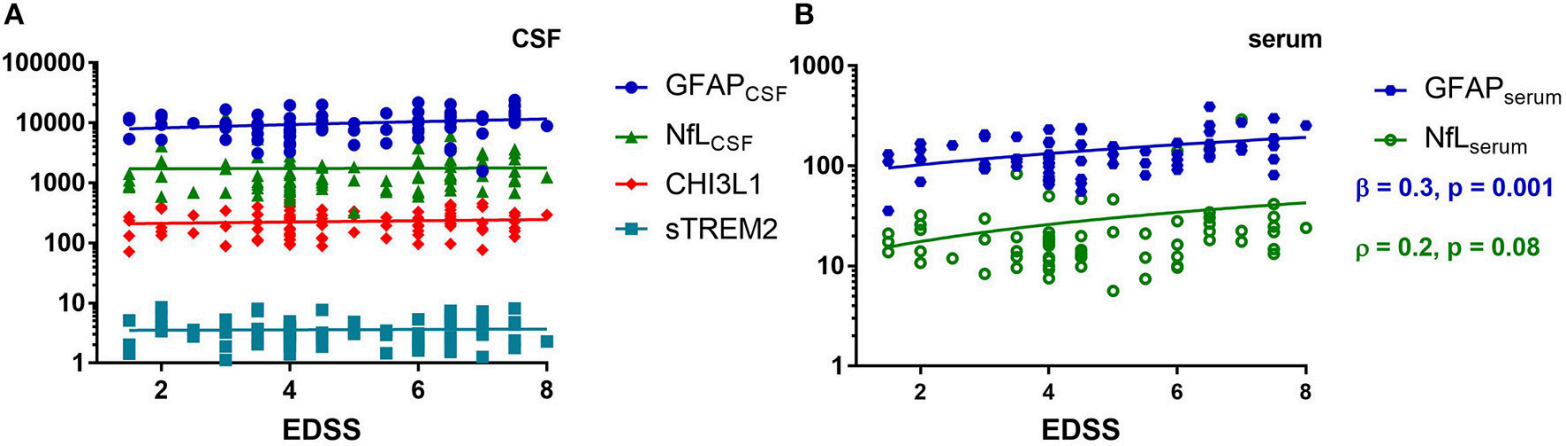
Spearman correlation of cerebrospinal fluid (CSF) **(A)** and serum **(B)** biomarkers with the clinical severity as measured by the expanded disability status scale (EDSS). Glial fibrillary acidic protein (GFAP), neurofilaments light chain (NfL), chitinase 3 like 1 (CHI3L1), soluble triggering receptor expressed on myeloid cells 2 (sTREM2), ρ (Spearman rho), β (standardized coefficient) of multiple linear regression, age as a covariant.

Regarding the serum markers, we found a moderate correlation between GFAP_serum_ and EDSS (ρ = 0.4, *p* = 0.004), which remained significant after adjusting by the effect of age (β = 0.3, *p* = 0.001) (Figure 3). NfL_serum_ did not correlate with any of the disease severity parameters (Figure 3). No significant correlations were found between GFAP_serum_ and MSSS or ARMSS (data not shown).

**Figure 4:**
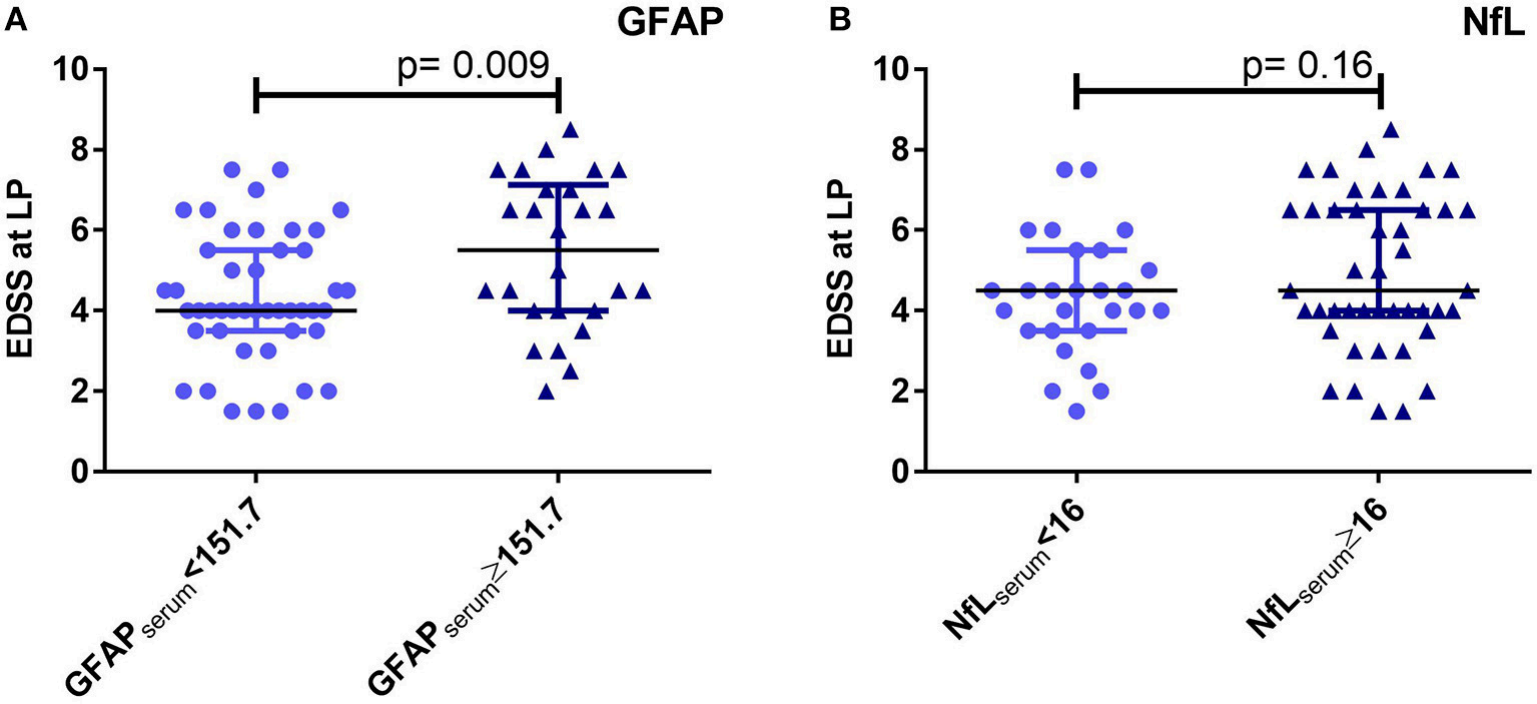
Comparison of expanded disability status scale (EDSS) values for primary progressive multiple sclerosis (PPMS) patients below and above cut-offs of 151.7 pg/ml for glial fibrillary acidic protein (GFAP) **(A)** and 16 pg/ml for neurofilaments light chain (NfL) **(B)** in serum (Mann-Whitney *U* test), respectively. There was a significant age difference for the grouping by NfL but not for GFAP (Mann-Whitney *U* test, *p* = 0.01 and 0.47, respectively). LP, lumbar puncture.

To further confirm the association of GFAP_serum_ with the EDSS, we compared the EDSS values with GFAP_serum_ and NfL_serum_ levels higher or lower than the cut-off that was determined as described beforehand. Here, 63% of our PPMS patients had GFAP_serum_ above this cut-off of 151.7 pg/ml (*n* = 45). Moreover, they had a significantly higher median EDSS than patients below this cut-off (5.5 vs. 4.0, *p* = 0.009, Figure 4). No differences were found for this comparison for NfL_serum_ (cut-off 16 pg/ml, 4.5 vs. 4.5, *p* = 0.16, Figure 4). There was a significant age difference for the grouping by NfL_serum_ but not for GFAP_serum_ (*p* = 0.01 and 0.47, respectively, data not shown).

**Figure 5:**
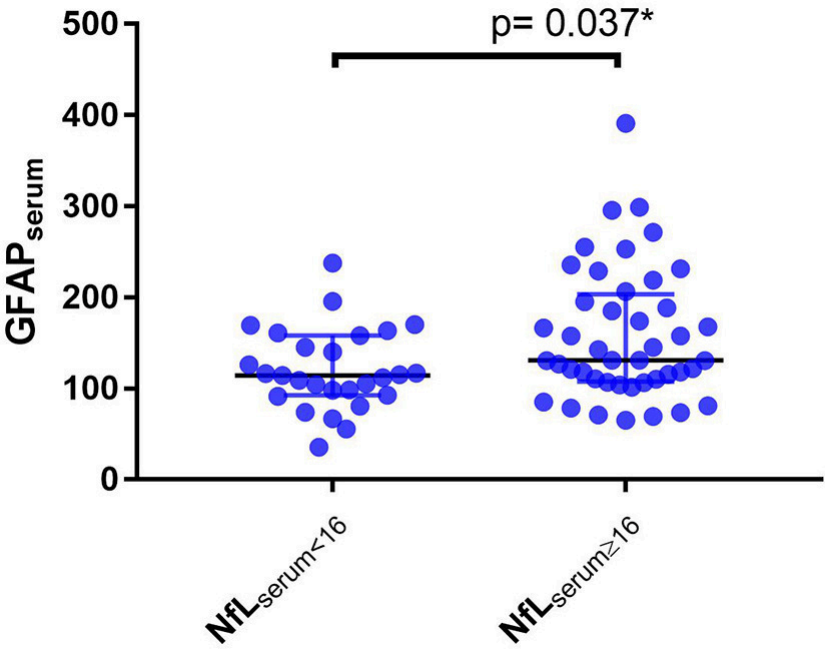
Level of serum glial fibrillary acidic protein (GFAP_serum_) in PPMS patients with serum neurofilaments light chain levels equal to or higher than 16 pg/ml (NfL_serum_ ≥ 16) or lower the 16 pg/ml (NfL_serum_ < 16), ^*^Univariant general linear model corrected for age.

Considering NfL_serum_ ≥ 16 pg/ml as a cut-off value for active diseases, PPMS patients with NfL_serum_ ≥ 16 pg/ml (*n* = 44) have higher median concentration of GFAP_serum_ than those with NfL_serum_ < 16 pg/ml (*n* = 27) (131.0 vs. 114.5 pg/ml, *p* = 0.037 after correction for age, Figure 5).

### Correlation Between CSF and Serum Parameters

We found a moderate correlation between GFAP levels in CSF and serum (ρ = 0.4, *p* = 0.001), and strong correlation between levels of NfL in CSF and serum (ρ = 0.6, *p* < 0.001).

**Figure 6:**
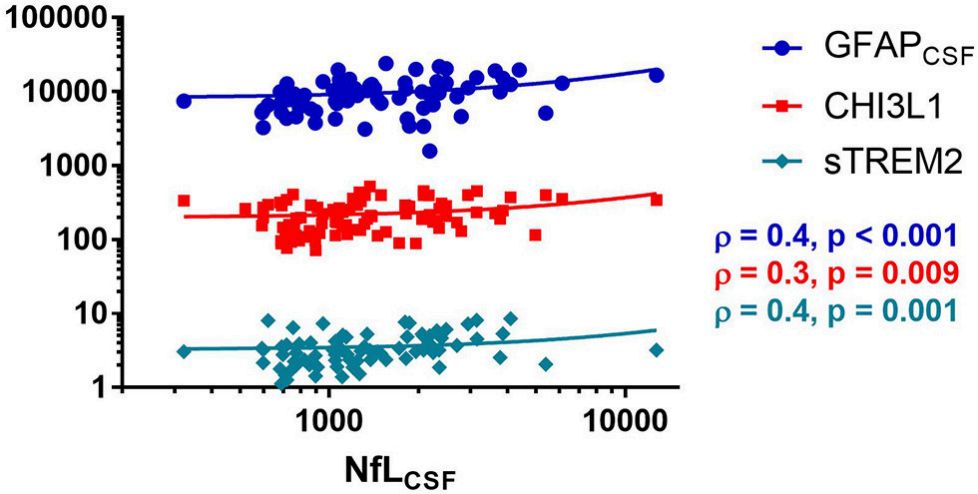
Spearman correlation between neurofilaments light chain (NfL), glial fibrillary acidic protein (GFAP), chitinase 3 like 1 (CHI3L1), and soluble triggering receptor expressed on myeloid cells 2 (sTREM2), ρ (Spearman rho).

**Figure 7:**
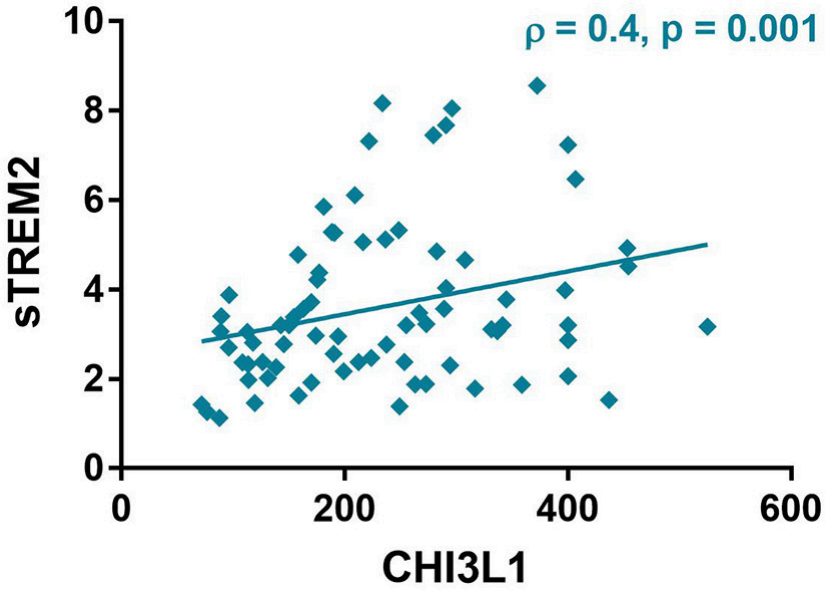
Spearman correlation between chitinase 3 like 1 (CHI3L1) and soluble triggering receptor expressed on myeloid cells 2 (sTREM2), ρ (Spearman rho).

In CSF, NfL correlated with sTREM2 (ρ = 0.4, *p* < 0.01,) CHI3L1 (ρ = 0.3, *p* < 0.01) and GFAP (ρ = 0.4, *p* < 0.001, Table 3 and Figure 6). Moreover, CHI3L1 correlated with sTREM2 (ρ = 0.4, *p* < 0.01, Figure 7) but not with GFAP. On the other hand, GFAP did not correlate with sTREM2.

**Table 3:** Correlations between various CSF biomarkers.

**Marker**	**NfL_**CSF**_**	**GFAP_**CSF**_**	**CHI3L1**
sTREM2	0.4^**^	n.s.	0.4^**^
CHI3L1	0.3^**^	n.s.	
GFAP_CSF_	0.4^***^		

*GFAP, glial fibrillary acidic protein; NfL, neurofilaments light chain; CHI3L1, chitinase 3 like 1; sTREM2, soluble triggering receptor expressed on myeloid cells 2*.

** *p < 0.01*,

*** *p < 0.001*.

## Discussion

Assessment of various biomarkers reflecting different pathophysiological processes involved in the disease progression and the downstream-treatment effect is becoming increasingly important due to the emerging treatment options for progressive MS. Our study evaluates the levels of astroglial activation (GFAP), microglial activation (CHI3L1 and sTREM2) and neuroaxonal damage (NfL) in CSF and serum of a multicentric cohort of PPMS-Patients.

The glial activation is a putative cornerstone in the progression of neurodegeneration in PPMS (1). The astrocytes involvement in MS generally is a double-edged sword; while the glial scar formation may protect the tissue from further damage, it might prevent the remyelination in MS (31). The activation of astrocytes is an early event in the development of MS lesions with the release of cytokines like CCL-2, CXCL-12, MMPs, TGFβ, IL-1, IL-6, IL-10, IL-12, IL-15, IL-23, and IL-27 leading to dysfunction of the blood-brain barrier (BBB) as well as recruitment of innate and adaptive immune cells (32). Furthermore, the A1 subtype of astrocytes is a potent killer of neurons and oligodendrocytes in EAE models and was reported in acute and chronic MS lesions (33).

Nevertheless, the astrocytes in progressive MS play a more specific role in the maintenance of the local inflammation; in models of chronic EAE, levels of lactosylceramide (LacCer) synthesized by β-1,4-galactosyltransferase 6 (B4GALT6) from the activated astrocytes were elevated in the EAE-lesions. Suppression of B4GALT6-activity reduces local inflammation, microglial activation and monocytes recruitment and subsequently the resulting neurodegeneration (34).

Correlation between GFAP_CSF_ and clinical severity were inconsistent among previous studies using the standard ELISA assay (9, 12–15). In a previous study from one of our centers, we reported a moderate correlation between levels of GFAP in serum, but not in CSF, with disease severity scores (16). Our current multicentric cohort validated our results showing a consistent correlation between GFAP_serum_ with EDSS score, even after correction for the age of the patients. In accordance with those results, patients with GFAP_serum_ above the proposed cut-off of 151.7 pg/ml based on our previous results, had more severe disease. Furthermore, levels with GFAP_serum_ were higher in patients having NfL_serum_ ≥ 16, a recently proposed cut-off value for higher activity and worse disease prognosis (30). Nevertheless, this NfL cut-off value was proposed according to pooled results from various studies with relapsing-remitting MS patients. Thus, its value in PPMS has yet to be validated.

Why GFAP_serum_ might reflect the disease activity better than GFAP_CSF_ is still not entirely explained. As previously suggested from our group, the enhanced expression of GFAP in the activated astrocytes end feet in the predominantly perivascular MS lesions might be directly drained into the blood compartment and not into the CSF space (16). Supporting data were reported in mouse models of EAE (35). Moreover, the GFAP, as well as other markers, might be transported to blood directly via the glymphatic system as shown recently in murine models of traumatic brain injury (36).

While a recent study suggested lower levels of GFAP_serum_ in relapsing MS patients under treatment (26), the concentration of GFAP_serum_ did not differ according to treatment status in our cohort. This can be explained by the fact, that none of the above-mentioned treatments were proven effective in PPMS. This observation regarding levels of GFAP_serum_, as well as other markers including the NfL_serum_, might underscore the ineffectiveness of those treatments in PPMS.

The validation of those results in our larger multicentric cohort might highlight the clinical meaning of levels of GFAP in serum as a possible serum marker in PPMS patients.

Like the astrocytes, the activated microglial release different cytokines (IL-1, Il-6, and TNF-α) as well as NO and ROS leading to exacerbation of inflammatory cascade, to attraction of inflammatory cells from blood and also to mitochondrial dysfunction, a significant mechanism in the disease progression in PMS (2, 3, 37, 38). Moreover, microglial activation in PMS appears to be diffusely prevalent not only in MS lesions but also in normal-appearing white matter (NAWM) forming the so-called microglial nodules. In the less inflammatory cortical lesions, active demyelination can be found in close proximity with the microglia (3).

Data regarding sTREM2 in PPMS are scarce with two studies reported concentration in CSF in twenty-one and in three PPMS patients, respectively (20, 22). In accordance with previous reports, no correlation was found between sTREM2 and EDSS or MSSS. Yet, sTREM2 correlated with NfL in CSF, which might highlight the role of microglia in the neuroaxonal demise in PPMS.

The meaning of CHI3L1 appears to be controversial; some reports consider it as a marker of astrocytic activation (18, 39), whereas other studies count it a marker of active microglial cells (40). The correlation between CHI3L1 and sTREM2 found in our patients is equivocal; it may underscore the microglial origin of CHI3L1 or might reflect the crosstalk between the microglia and astrocytes in PPMS (33) leaving the question regarding the origin of CHI3L1 in MS brains unanswered.

The prognostic value of CHI3L1 is prominent in CIS and RRMS patients (13, 17, 41–44) and to a lesser extent in SPMS (19). CHI3L1 in CSF did not correlate with the clinical parameters in PPMS patients in the above-mentioned studies. Consistent with the various histopathological studies, all measured glial activation markers in CSF correlated with the neuroaxonal demise as assessed by the NfL, but not with the clinical severity scores. This paradox might be due to some methodological limitations of the assays, the need of more specific markers or due to the limited ability of the applied clinical scores to reflect the extent of the neurodegeneration in PPMS. Indeed, the shortness of EDSS to reflect all aspects of the disease progression in PPMS is a lesson learned from the various negative clinical trials in PPMS (45). The upper arm function and the subtle cognitive deficits are underrepresented in the EDSS (46). Furthermore, the EDSS scores were mostly based on the walking distance reported by the patients, which might lead to an incorrect evaluation of the EDSS score (47).

Another limitation of our study is the missing detailed magnetic resonance imaging data (MRI). However, the main aim of our study was to explore the clinical meaning of the glial activation markers.

Although the concentration of most of the measured biomarkers did not vary between centers, GFAP and sTREM2 were notably lower in two of the four centers. As this is a retrospective study, we were not able to completely capture all pre-analytical procedures. To avoid potential bias of pre-analytical procedures, we did the comparison of analyte levels per center and excluded statistically low values. The excluded samples represent a minority in our cohort (17/93 for GFAP and 6/93 for sTREM2), and their exclusion had no statistical impact on the above-mentioned results (data not shown).

A point to be considered while interpreting our results is the protective role of glial cells following neuronal injury. Microglial and astrocytic activation were reported upon axonal degeneration and contributes to tissue repair and limitation of the inflammatory activity (48–50). Our markers reflect the glial activation generally, but not necessarily their pathological role. Complementary data from more specific markers of subsets of microglia or astrocytes could be helpful to understand their pathogenetic role.

In summary, we analyzed in this study various markers of glial activation in CSF and serum and evaluated their correlation with neuroaxonal damage markers and disease severity measures. The correlation between the microglial and astrocytic activation markers in CSF with the markers of neuroaxonal demise (NfL) may underscore the glial involvement in the neurodegeneration in PPMS. The positive correlation between GFAP in serum with the clinical severity of the disease may highlight the potential of GFAP_serum_ as a disease progression marker. It his highly desirable to confirm this finding in a prospectively collected study cohort and compare it to standardized acquired MRI data. Additionally, the determination of GFAP serum levels in a large group of healthy controls might help to further differentiate between age related normal and abnormal GFAP levels.

## Data Availability

The datasets generated for this study are available on request to the corresponding author.

## Ethics Statement

The study was reviewed by the ethics committee of the University of Ulm, and all experimental protocols were approved (approval number 270/17). Our study was performed in accordance with the ethical standards of the 1964 Declaration of Helsinki. Written informed consent was obtained from all patients participating in this study.

## Author Contributions

AA, HT, and AH: study concept. AA and TH: data acquisition, data analysis, and interpretation. AH, EM-R, and MS-C: biomarkers assessment. AA: drafting of the manuscript. HT, SR, MO, CH, UZ, and SM: study supervision and critical revision. All authors critically reviewed and approved the manuscript.

### Conflict of Interest Statement

The authors declare that the research was conducted in the absence of any commercial or financial relationships that could be construed as a potential conflict of interest.

## References

[1] Abdelhak A Weber MS Tumani H. Primary progressive multiple sclerosis: putting together the puzzle. Front Neurol. (2017) 8:234. 10.3389/fneur.2017.00234 28620346 PMC5449443

[2] Mahad DH Trapp BD Lassmann H. Pathological mechanisms in progressive multiple sclerosis. Lancet Neurol. (2015) 14:183–93. 10.1016/S1474-4422(14)70256-X 25772897

[3] Lassmann H van Horssen J Mahad D. Progressive multiple sclerosis: pathology and pathogenesis. Nat Rev Neurol. (2012) 8:647–56. 10.1038/nrneurol.2012.168 23007702

[4] Lassmann H. Mechanisms of white matter damage in multiple sclerosis. Glia. (2014) 62:1816–30. 10.1002/glia.22597 24470325

[5] Abdelhak A Junker A Brettschneider J Kassubek J Ludolph AC Otto M. Brain-specific cytoskeletal damage markers in cerebrospinal fluid: is there a common pattern between amyotrophic lateral sclerosis and primary progressive multiple sclerosis? Int J Mol Sci. (2015) 16:17565–88. 10.3390/ijms160817565 26263977 PMC4581209

[6] Tumani H Teunissen C Sussmuth S Otto M Ludolph AC Brettschneider J. Cerebrospinal fluid biomarkers of neurodegeneration in chronic neurological diseases. Expert Rev Mol Diagn. (2008) 8:479–94. 10.1586/14737159.8.4.479 18598229

[7] Eng LF. Glial fibrillary acidic protein (GFAP): the major protein of glial intermediate filaments in differentiated astrocytes. J Neuroimmunol. (1985) 8:203–14. 10.1016/S0165-5728(85)80063-1 2409105

[8] Rosengren LE Lycke J Andersen O. Glial fibrillary acidic protein in CSF of multiple sclerosis patients: relation to neurological deficit. J Neurol Sci. (1995) 133:61–5. 10.1016/0022-510X(95)00152-R 8583233

[9] Petzold A Eikelenboom MJ Gveric D Keir G Chapman M Lazeron RH. Markers for different glial cell responses in multiple sclerosis: clinical and pathological correlations. Brain. (2002) 125 (Pt 7):1462–73. 10.1093/brain/awf165 12076997

[10] Malmestrom C Haghighi S Rosengren L Andersen O Lycke J. Neurofilament light protein and glial fibrillary acidic protein as biological markers in MS. Neurology. (2003) 61:1720–5. 10.1212/01.WNL.0000098880.19793.B6 14694036

[11] Linker RA Brechlin P Jesse S Steinacker P Lee DH Asif AR. Proteome profiling in murine models of multiple sclerosis: identification of stage specific markers and culprits for tissue damage. PLoS ONE. (2009) 4:e7624. 10.1371/journal.pone.0007624 19865482 PMC2765069

[12] Axelsson M Malmestrom C Nilsson S Haghighi S Rosengren L Lycke J. Glial fibrillary acidic protein: a potential biomarker for progression in multiple sclerosis. J Neurol. (2011) 258:882–8. 10.1007/s00415-010-5863-2 21197541

[13] Martinez MA Olsson B Bau L Matas E Cobo Calvo A Andreasson U. Glial and neuronal markers in cerebrospinal fluid predict progression in multiple sclerosis. Mult Scler. (2015) 21:550–61. 10.1177/1352458514549397 25732842 PMC4390605

[14] Mane-Martinez MA Olsson B Bau L Matas E Cobo-Calvo A Andreasson U. Glial and neuronal markers in cerebrospinal fluid in different types of multiple sclerosis. J Neuroimmunol. (2016) 299:112–7. 10.1016/j.jneuroim.2016.08.004 27725108

[15] Kassubek R Gorges M Schocke M Hagenston VAM Huss A Ludolph AC. GFAP in early multiple sclerosis: a biomarker for inflammation. Neurosci Lett. (2017) 657:166–70. 10.1016/j.neulet.2017.07.050 28802830

[16] Abdelhak A Huss A Kassubek J Tumani H Otto M. Serum GFAP as a biomarker for disease severity in multiple sclerosis. Sci Rep. (2018) 8:14798. 10.1038/s41598-018-33158-8 30287870 PMC6172254

[17] Hinsinger G Galeotti N Nabholz N Urbach S Rigau V Demattei C. Chitinase 3-like proteins as diagnostic and prognostic biomarkers of multiple sclerosis. Mult Scler. (2015) 21:1251–61. 10.1177/1352458514561906 25698171

[18] Querol-Vilaseca M Colom-Cadena M Pegueroles J San Martin-Paniello C Clarimon J Belbin O. YKL-40 (Chitinase 3-like I) is expressed in a subset of astrocytes in Alzheimer's disease and other tauopathies. J Neuroinflammation. (2017) 14:118. 10.1186/s12974-017-0893-7 28599675 PMC5466718

[19] Burman J Raininko R Blennow K Zetterberg H Axelsson M Malmestrom C. YKL-40 is a CSF biomarker of intrathecal inflammation in secondary progressive multiple sclerosis. J Neuroimmunol. (2016) 292:52–7. 10.1016/j.jneuroim.2016.01.013 26943959

[20] Piccio L Buonsanti C Cella M Tassi I Schmidt RE Fenoglio C. Identification of soluble TREM-2 in the cerebrospinal fluid and its association with multiple sclerosis and CNS inflammation. Brain. (2008) 131 (Pt 11):3081–91. 10.1093/brain/awn217 18790823 PMC2577803

[21] Kleinberger G Yamanishi Y Suarez-Calvet M Czirr E Lohmann E Cuyvers E. TREM2 mutations implicated in neurodegeneration impair cell surface transport and phagocytosis. Sci Transl Med. (2014) 6:243ra86. 10.1126/scitranslmed.3009093 24990881

[22] Ohrfelt A Axelsson M Malmestrom C Novakova L Heslegrave A Blennow K Soluble TREM-2 in cerebrospinal fluid from patients with multiple sclerosis treated with natalizumab or mitoxantrone. Mult Scler. (2016) 22:1587–95. 10.1177/1352458515624558 26754805

[23] Suarez-Calvet M Kleinberger G Araque Caballero MA Brendel M Rominger A Alcolea D. sTREM2 cerebrospinal fluid levels are a potential biomarker for microglia activity in early-stage Alzheimer's disease and associate with neuronal injury markers. EMBO Mol Med. (2016) 8:466–76. 10.15252/emmm.201506123 26941262 PMC5120370

[24] Kuhle J Barro C Disanto G Mathias A Soneson C Bonnier G. Serum neurofilament light chain in early relapsing remitting MS is increased and correlates with CSF levels and with MRI measures of disease severity. Mult Scler. (2016) 22:1550–9. 10.1177/1352458515623365 26754800

[25] Bogoslovsky T Wilson D Chen Y Hanlon D Gill J Jeromin A. Increases of Plasma Levels of Glial Fibrillary Acidic Protein, Tau, and Amyloid beta up to 90 Days after Traumatic Brain Injury. J Neurotrauma. (2017) 34:66–73. 10.1089/neu.2015.4333 27312416 PMC5198034

[26] Hogel H Rissanen E Barro C Matilainen M Nylund M Kuhle J. Serum glial fibrillary acidic protein correlates with multiple sclerosis disease severity. Mult Scler. (2018). 10.1177/1352458518819380 30570436

[27] Thompson AJ Banwell BL Barkhof F Carroll WM Coetzee T Comi G. Diagnosis of multiple sclerosis: 2017 revisions of the McDonald criteria. Lancet Neurol. (2017) 17:162–173. 10.1016/S1474-4422(17)30470-2 29275977

[28] Manouchehrinia A Westerlind H Kingwell E Zhu F Carruthers R Ramanujam R. Age related multiple sclerosis severity score: disability ranked by age. Mult Scler. (2017) 23:1938–46. 10.1177/1352458517690618 28155580 PMC5700773

[29] Teunissen CE Petzold A Bennett JL Berven FS Brundin L Comabella M. A consensus protocol for the standardization of cerebrospinal fluid collection and biobanking. Neurology. (2009) 73:1914–22. 10.1212/WNL.0b013e3181c47cc2 19949037 PMC2839806

[30] Calabresi P Kuhle J Arnold DL Singh CM Kinkel RP Kappos L Serum Neurofilament Light (NfL) for Disease Prognosis and Treatment Monitoring in Multiple Sclerosis Patients: Is It Ready for Implementation into Clinical Care? Berlin: European Committee for Treatment and Research in Multiple Sclerosis (ECTRIMS) (2018).

[31] Holley JE Gveric D Newcombe J Cuzner ML Gutowski NJ. Astrocyte characterization in the multiple sclerosis glial scar. Neuropathol Appl Neurobiol. (2003) 29:434–44. 10.1046/j.1365-2990.2003.00491.x 14507335

[32] Mayo L Quintana FJ Weiner HL. The innate immune system in demyelinating disease. Immunol Rev. (2012) 248:170–87. 10.1111/j.1600-065X.2012.01135.x 22725961 PMC3383669

[33] Liddelow SA Guttenplan KA Clarke LE Bennett FC Bohlen CJ Schirmer L. Neurotoxic reactive astrocytes are induced by activated microglia. Nature. (2017) 541:481–7. 10.1038/nature21029 28099414 PMC5404890

[34] Mayo L Trauger SA Blain M Nadeau M Patel B Alvarez JI. Regulation of astrocyte activation by glycolipids drives chronic CNS inflammation. Nat Med. (2014) 20:1147–56. 10.1038/nm.3681 25216636 PMC4255949

[35] Sofroniew MV Vinters HV. Astrocytes: biology and pathology. Acta Neuropathol. (2010) 119:7–35. 10.1007/s00401-009-0619-8 20012068 PMC2799634

[36] Plog BA Dashnaw ML Hitomi E Peng W Liao Y Lou N. Biomarkers of traumatic injury are transported from brain to blood via the glymphatic system. J Neurosci. (2015) 35:518–26. 10.1523/JNEUROSCI.3742-14.2015 25589747 PMC4293408

[37] Zrzavy T Hametner S Wimmer I Butovsky O Weiner HL Lassmann H. Loss of 'homeostatic' microglia and patterns of their activation in active multiple sclerosis. Brain. (2017) 140:1900–13. 10.1093/brain/awx113 28541408 PMC6057548

[38] Lassmann H. Multiple sclerosis: lessons from molecular neuropathology. Exp Neurol. (2014) 262 (Pt A):2–7. 10.1016/j.expneurol.2013.12.003 24342027

[39] Bonneh-Barkay D Wang G Starkey A Hamilton RL Wiley CA. In vivo CHI3L1 (YKL-40) expression in astrocytes in acute and chronic neurological diseases. J Neuroinflammation. (2010) 7:34. 10.1186/1742-2094-7-34 20540736 PMC2892443

[40] Michelucci A Heurtaux T Grandbarbe L Morga E Heuschling P. Characterization of the microglial phenotype under specific pro-inflammatory and anti-inflammatory conditions: effects of oligomeric and fibrillar amyloid-beta. J Neuroimmunol. (2009) 210:3–12. 10.1016/j.jneuroim.2009.02.003 19269040

[41] Comabella M Fernandez M Martin R Rivera-Vallve S Borras E Chiva C. Cerebrospinal fluid chitinase 3-like 1 levels are associated with conversion to multiple sclerosis. Brain. (2010) 133 (Pt 4):1082–93. 10.1093/brain/awq035 20237129

[42] Canto E Tintore M Villar LM Costa C Nurtdinov R Alvarez-Cermeno JC. Chitinase 3-like 1: prognostic biomarker in clinically isolated syndromes. Brain. (2015) 138 (Pt 4):918–31. 10.1093/brain/awv017 25688078

[43] Quintana E Coll C Salavedra-Pont J Munoz-San Martin M Robles-Cedeno R Tomas-Roig J. Cognitive impairment in early stages of multiple sclerosis is associated with high cerebrospinal fluid levels of chitinase 3-like 1 and neurofilament light chain. Eur J Neurol. (2018) 25:1189–91. 10.1111/ene.13687 29797629

[44] Sellebjerg F Royen L Soelberg Sorensen P Oturai AB Jensen PEH. Prognostic value of cerebrospinal fluid neurofilament light chain and chitinase-3-like-1 in newly diagnosed patients with multiple sclerosis. Mult Scler. (2018). 10.1177/1352458518794308 30113249

[45] Ontaneda D Fox RJ Chataway J. Clinical trials in progressive multiple sclerosis: lessons learned and future perspectives. Lancet Neurol. (2015) 14:208–23. 10.1016/S1474-4422(14)70264-9 25772899 PMC4361791

[46] Tur C Moccia M Barkhof F Chataway J Sastre-Garriga J Thompson AJ. Assessing treatment outcomes in multiple sclerosis trials and in the clinical setting. Nat Rev Neurol. (2018) 14:75–93. 10.1038/nrneurol.2017.171 29326424

[47] Skjerbaek AG Boesen F Petersen T Rasmussen PV Stenager E Norgaard M. Can we trust self-reported walking distance when determining EDSS scores in patients with multiple sclerosis? The Danish MS hospitals rehabilitation study. Mult Scler. (2018). 10.1177/1352458518795416 30124106

[48] Belanger M Magistretti PJ. The role of astroglia in neuroprotection. Dialogues Clin Neurosci. (2009) 11:281–95. 19877496 10.31887/DCNS.2009.11.3/mbelanger PMC3181926

[49] Neumann H Kotter MR Franklin RJ. Debris clearance by microglia: an essential link between degeneration and regeneration. Brain. (2009) 132 (Pt 2):288–95. 10.1093/brain/awn109 18567623 PMC2640215

[50] Suarez-Calvet M Araque Caballero MA Kleinberger G Bateman RJ Fagan AM Morris JC. Early changes in CSF sTREM2 in dominantly inherited Alzheimer's disease occur after amyloid deposition and neuronal injury. Sci Transl Med. (2016) 8:369ra178. 10.1126/scitranslmed.aag1767 27974666 PMC5385711

